# The Cryptic Nature of Fe-S Clusters: A Case Study of the Hepatitis B HBx Oncoprotein

**DOI:** 10.3390/inorganics11120475

**Published:** 2023-12-06

**Authors:** Trent Quist, Jiahua Chen, Alex MacNeil, Maria-Eirini Pandelia

**Affiliations:** Department of Biochemistry, Brandeis University, Waltham, MA 02453, USA;

**Keywords:** Fe-S clusters, EPR spectroscopy, Mössbauer spectroscopy, UV/VIS, Hepatitis B

## Abstract

Fe-S clusters are ubiquitous inorganic cofactors found in proteins across all domains of life, including viruses. Their prevalence stems from their unique redox and structural plasticity that supports functions ranging from electron transfer and catalysis to stabilization of protein structure. Although the ability of Fe-S clusters to exchange electrons is often functionally crucial, it can also act as an Achilles heel when these cofactors are exposed to oxidizing conditions, often leading to their degradation. This O_2_ sensitivity has rendered certain Fe-S clusters untraceable, particularly when the nascent proteins are isolated under ambient conditions. As a consequence of this O_2_ sensitivity, a growing number of proteins with roles in viral infection have been found to harbor Fe-S clusters rather than the annotated Zn^2+^ cofactor. The enigmatic protein X (HBx) of the Hepatitis B Virus is a multifunctional protein essential for viral replication and development of liver disease. Although HBx has defied biochemical characterization for over forty years, it has been shown to coordinate a redox-active Fe-S cluster that represents a significant feature for establishing its molecular function. The present review narrates the approaches to validate the HBx metallocofactor that can be broadly applied as a guide for uncovering the presence of Fe-S clusters in proteins with non-canonical sequence motifs.

## 1. Introduction

Fe-S clusters are evolutionarily ancient inorganic cofactors that occur in proteins from all domains of life and viruses, to which they endow a remarkable structural and functional diversity [1–5]. Fe-S clusters consist of iron (Fe) and inorganic sulfide (S^2−^) with the redox-active Fe ions in a tetrahedral geometry attained by S^2−^ and protein-based ligands. The ability of Fe ions to undergo redox chemistry, together with the extraordinary plasticity of Fe-S clusters, have marked these cofactors as the preferred mediators in electron transfer reactions. Fe-S clusters, however, are remarkably multifunctional, and in addition to their ability to exchange electrons, they also serve as structural elements, catalytic centers, small molecule sensors (i.e., O_2_, NO) and donors (i.e., iron and sulfide), and electron carriers to or recruiters of nucleic acids [2,3,6–10].

While classical Fe-S clusters are coordinated by cysteine and/or histidine amino acid residues, identification of their presence on the basis of a sequence is ambiguous due to the expanding repertoire of non-canonical binding motifs that include variable spacings of these and other residues (e.g., aspartate, asparagine) [11,12]. In addition, a number of factors make the discovery of Fe-S clusters uniquely challenging. These metalloclusters are inherently redox-sensitive with high reactivity towards molecular O_2_, partially reduced oxygen species, nitric oxide, as well as many oxidizing and reducing agents [8,13]. Isolation of Fe-S cluster proteins under ambient conditions often leads to oxidative degradation of Fe-S clusters that renders them intractable. The absence of an overexpressed Fe-S cluster biosynthesis machinery during heterologous expression may also contribute to the absence of these cofactors in purified proteins [14–16]. Because the metallocofactor sites are primarily made up of cysteine and/or histidine residues, they exhibit high promiscuity in coordinating other divalent metal ions, with the Zn^2+^ ion being the most frequently observed binding proxy [17–19]. The growing number of Fe-S cluster proteins that interchangeably coordinate both Fe-S clusters and Zn ions, however, makes identification of the bona fide cofactor cryptic and often leads to their erroneous assignment as Zinc-Finger (ZF) proteins [18,20–22].

Recently, several medically and biotechnologically relevant viruses have been found to encode putative ZF proteins that are recognized to participate in antiviral defense, replication, and regulation of the viral life cycle [21–24]. Their inference as Zn binding proteins is based on the presence of a CCCC or CCCH motif and, in a few cases, on crystallographic studies. Interestingly, some putative viral ZF proteins have been experimentally demonstrated to bind and utilize an Fe-S cluster in vivo. Nsp12, the catalytic subunit of the SARS-CoV-2 RNA-dependent polymerase, is an annotated ZF protein required for transcription and viral replication [22]. Seminal studies by Rouault and coworkers established that Nsp12 coordinates two [4Fe-4S] clusters that are provided by the host ISC machinery [22]. The same group also reported that the helicase Nsp13 coordinates a [4Fe-4S] cluster in the putative Zn-binding domain that is important for RNA binding and unwinding activity [21]. The Hepatitis B viral oncoprotein HBx was also initially annotated to coordinate Zn by a CCCH motif, while later studies demonstrated that HBx coordinates either a [2Fe-2S] or a [4Fe-4S] cluster [23]. The Oncogenic Small Tumor Antigen of the Merkel Cell Polyomavirus (MCPyV) was also thought to coordinate two Zn^2+^ ions, which were later spectroscopically demonstrated to be [2Fe-2S] and [4Fe-4S] clusters, respectively [24], instead. Fe-S clusters have also been discovered in the small protein R633b from the *Megavirinae* giant viruses, albeit with an unrecognized role [25].

Undisputedly, Fe-S clusters are cofactors of unacknowledged function in viral proteins and are markedly more prevalent than initially considered. Their presence in vivo is assumed to rely on the host Fe-S cluster biosynthetic machinery, suggesting that the viral proteins are recognized as Fe-S cluster recipients. One of the mechanisms by which the host Fe-S scaffold and chaperone proteins are guided for Fe-S cluster delivery is the presence of a leucine–tyrosine–arginine (LYR) tripeptide on the viral protein [22,26]. However, other recognition processes must exist in the cell depending on the cellular localization of these proteins. Nevertheless, the growing number of Fe-S clusters discovered in viral proteins offers a paradox and numerous questions about the role of these cofactors in the host/virus interaction. The present review collects the core strategies that can be broadly applied for fingerprinting Fe-S cofactors in viral (and other) proteins, irrespective of their primary sequence features, and narrates the events for the identification of the Fe-S cluster in HBx from HBV.

## 2. Methods for Fingerprinting Fe-S Clusters

### 2.1. UV/VIS Spectroscopy

**Figure 1.**
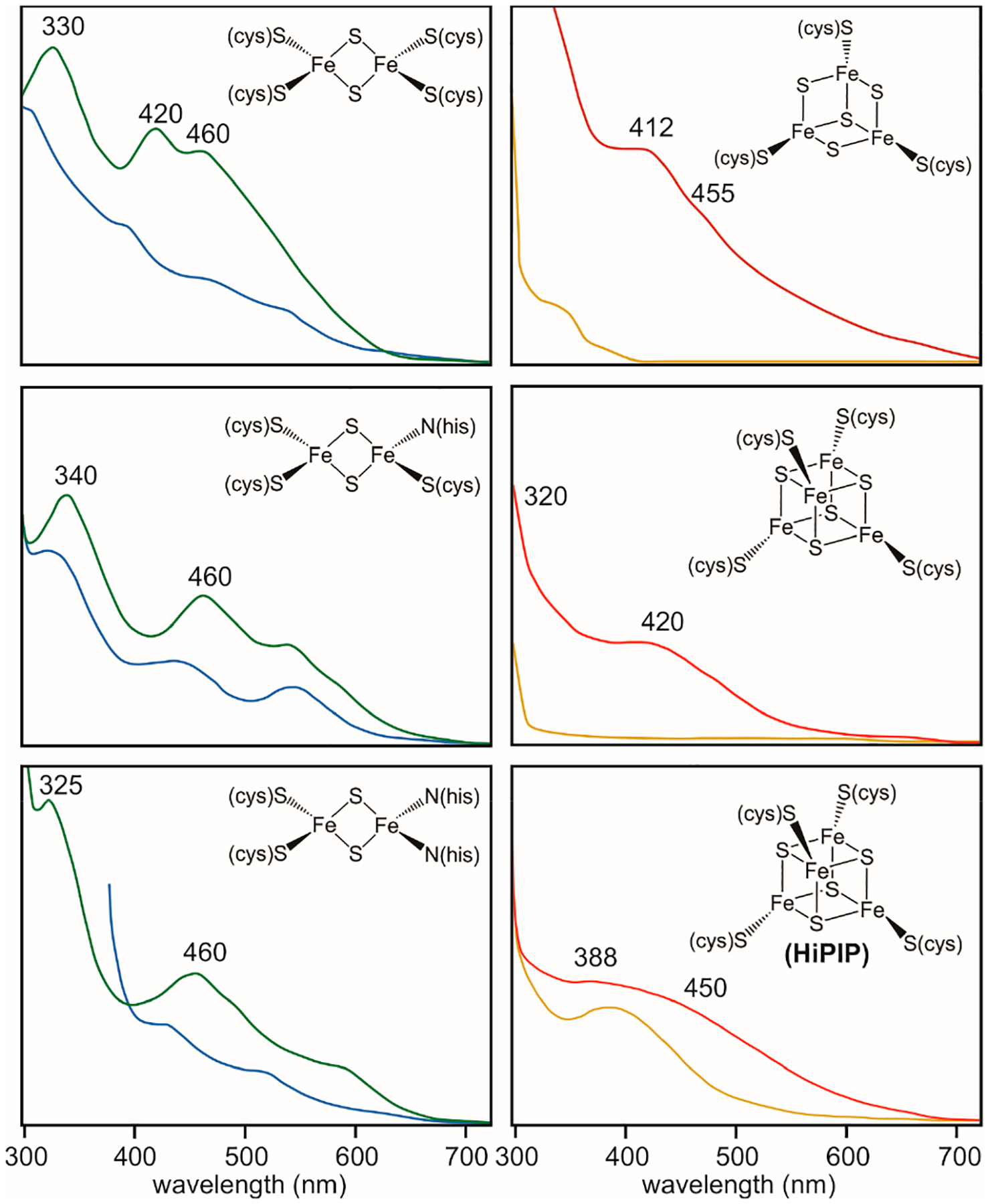
Characteristic UV/VIS spectra of [2Fe-2S], [3Fe-4S], and [4Fe-4S] clusters. The oxidized [2Fe-2S]^2+^ clusters are shown in green, and the one electron reduced forms in light blue. The spectra of the oxidized [3Fe-4S]^1+^, [4Fe-4S]^2+^, and [4Fe-4S]^3+^ forms are shown in red; the spectra of their one-electron reduced forms are shown in yellow. The spectra have been adapted from references [27–41].

Optical spectroscopy has played a central role in the characterization of Fe-S clusters, as these cofactors exhibit bands within the visible region of the electromagnetic spectrum, endowing their native proteins with a brown or reddish color. The absorption bands originate from ligand-to-metal charge transfer (LMCT) transitions that differ depending on the type of Fe-S cluster, its protein ligands, and its formal redox state [27,28]. While unambiguous identification of Fe-S clusters is challenging due to the (i) complexity of the electronic transitions, (ii) broadness of absorption bands, and (iii) variability in extinction coefficients, UV/VIS spectroscopy offers a simple and accessible diagnostic tool for inferring their presence and type. As a general rule, the oxidized forms of Fe-S clusters display the most pronounced optical features, while the reduced forms exhibit broader and less intense bands (Figure 1) [27,29–31]. The chemical nature of Fe-S cluster ligands can influence the optical properties of Fe-S clusters, giving rise to distinguishable spectra from which the type and overall oxidation state can be inferred.

#### 2.1.1. [2Fe-2S] Clusters

All-thiolate ligated [2Fe-2S]^2+^ clusters exhibit three bands at approximately 330 nm, 420 nm, and 460 nm [32,33]. Replacement of one of the cysteines with a histidine (CCCH) in MitoNEET and related proteins or two of the cysteines with histidines (CCHH) in Rieske-type proteins results in detectable spectral changes [32,34–38]. CCCH [2Fe-2S]^2+^ clusters display absorption maxima at 340 nm and 460 nm, accompanied by less intense peaks around 535 nm and 580 nm. CCHH [2Fe-2S]^2+^ clusters display maxima at 325 nm and 460 nm, as well as a shoulder at ~560–580 nm. An increase in the number of histidine coordinations results in spectral shifts towards longer wavelengths and the loss of the two-main-peak character and has been observed both with native and peptide ligands [33]. The LMCT bands appear to be diagnostic of the presence of an atypical ligation, in particular of a histidine ligand, but other coordination changes may not be detectable. The IscU scaffold protein, in which one of the Fe sites is coordinated by an aspartate, exhibits a spectrum that closely resembles that of four-cysteine ligated [2Fe-2S]^2+^ clusters, suggesting that the absence of unusual LMCT bands does not exclude an atypical ligation pattern [14]. The one-electron reduced [2Fe-2S]^1+^ state retains the overall spectral features, albeit with a ~50% reduction in intensity [27]. In some spectra of [2Fe-2S]^1+^ forms an additional band at 540 nm is sometimes observed, likely originating from asymmetry changes in electronic distribution introduced by the histidine ligand [32–34].

#### 2.1.2. [3Fe-4S] Clusters

The optical spectra of both cuboidal and linear [3Fe-4S]^1+^ clusters exhibit broad features with a maximum at 412 nm and less distinct shoulders at 320 nm and 455 nm (Figure 1) [27,32,39]. The one-electron reduced [3Fe-4S]^0^ state generally shows a peak at 425 nm, demonstrating that these two oxidation forms can be spectroscopically differentiated.

#### 2.1.3. [4Fe-4S] Clusters

The optical spectra of [4Fe-4S]^2+^ clusters typically feature a broad band centered between 380 and 420 nm, with a small shoulder near 305 nm (Figure 1) [14,27]. (The spectral features of [4Fe-4S]^2+^ clusters are broad and generally do not allow for inference of non-cysteinyl ligands as the exerted changes are hidden in the spectral envelope. As such, radical SAM (RS) enzymes, in which the catalytic [4Fe-4S] cluster is ligated by three cysteines and an unknown small molecule or solvent ligand, exhibit spectra reminiscent of all-thiolate ligated clusters [40]. The spectrum of the one-electron oxidized [4Fe-4S]^3+^ cluster exhibits spectra with a maximum at 388 nm and a shoulder around 450 nm [27,41]. On the other hand, one-electron reduction of the [4Fe-4S]^2+^ state results in the loss of the spectroscopic features, rendering [4Fe-4S]^1+^ clusters optically transparent [27].

### 2.2. Electron Paramagnetic Resonance (EPR) Spectroscopy

EPR probes the paramagnetic states of Fe-S centers [42,43]. In the presence of an external magnetic field (B_0_), the originally degenerate electronic spin states split due to the Zeeman interaction, and the magnitude of this effect depends on the strength of B_0_, the Bohr magneton (μ_B_), and the effective g-value, according to the equation: ΔE = hν = g·μ_B_·B_0_. The g-value of an electron bound to an atom is different from the free-electron g-value due to contributions of angular momentum. This effective g-value reflects the composite electronic environment of a Fe-S cluster, in which the unpaired spin is greatly delocalized. Therefore, g-values can be used as fingerprints to identify the type of a Fe-S cluster and any changes in its local structure. EPR signals consist of up to three g-values due to the spatial non-uniformity of the magnetic field produced by the electron cloud of the molecule. The signals can thus be classified as isotropic (g_x_ = g_y_ = g_z_), axial (g_x_ = g_y_ ≠ g_z_), or rhombic (g_x_ ≠ g_y_ ≠ g_z_) [43].

Historically, EPR and Fe-S clusters have been intimately connected. The first-ever observation of a Fe-S cluster was made in 1960 by Beinert et al. in succinate and DPNH dehydrogenase, in which an unusual signal at g = 1.94 was observed that could not be attributed to any known Fe-containing species [44]. Six years later, Gibson et al. proposed the theory and exchange model between the two Fe ions in spinach ferredoxin that enabled an understanding of the electronic structure of Fe-S clusters and set the stage for utilizing continuous wave (CW) EPR for the study of these metalloclusters [45]. Because transition metal complexes have short electron relaxation times, measurements are carried out at low temperatures, typically between 4.2–200 K. While g-values and g-anisotropy are good criteria for the identification of the nuclearity of a Fe-S cluster, the electron spin relaxation properties can offer an additional recognition trait. Overall, a good approximation is that Fe-S clusters with a higher number of Fe ions exhibit faster relaxation rates, owing to the larger density of excited states and relaxation pathways [46]. CW EPR has proven to be a powerful tool for interrogating the geometric and electronic structure of Fe-S clusters, as well as any conformational changes or substrate binding events [29,42,43,47]. The present review focuses on the three most commonly occurring polynuclear Fe-S clusters: [2Fe-2S], [3Fe-4S], and [4Fe-4S] clusters (Table 1).

#### 2.2.1. [2Fe-2S] Clusters

The oxidized [2Fe-2S]^2+^ form consists of two antiferromagnetically (AF)-coupled Fe^3+^ ions and has a diamagnetic (S = 0) ground state that is EPR-silent. The one-electron reduced [2Fe-2S]^1+^ form consists of a valence-trapped Fe^2+^-Fe^3+^ pair that has an S = 1/2 ground state and is EPR-active [1,42,76]. [2Fe-2S]^1+^ clusters often show the slowest relaxation rates among all other iron-sulfur centers and are usually best observed at ~40 K, while in some cases, they can be detected at up to 200 K [29,34,76]. Protein conformational heterogeneity, however, can result in fast relaxing [2Fe-2S] clusters, making the assignment of cluster type solely based on the relaxation properties ambiguous.

**Figure 2.**
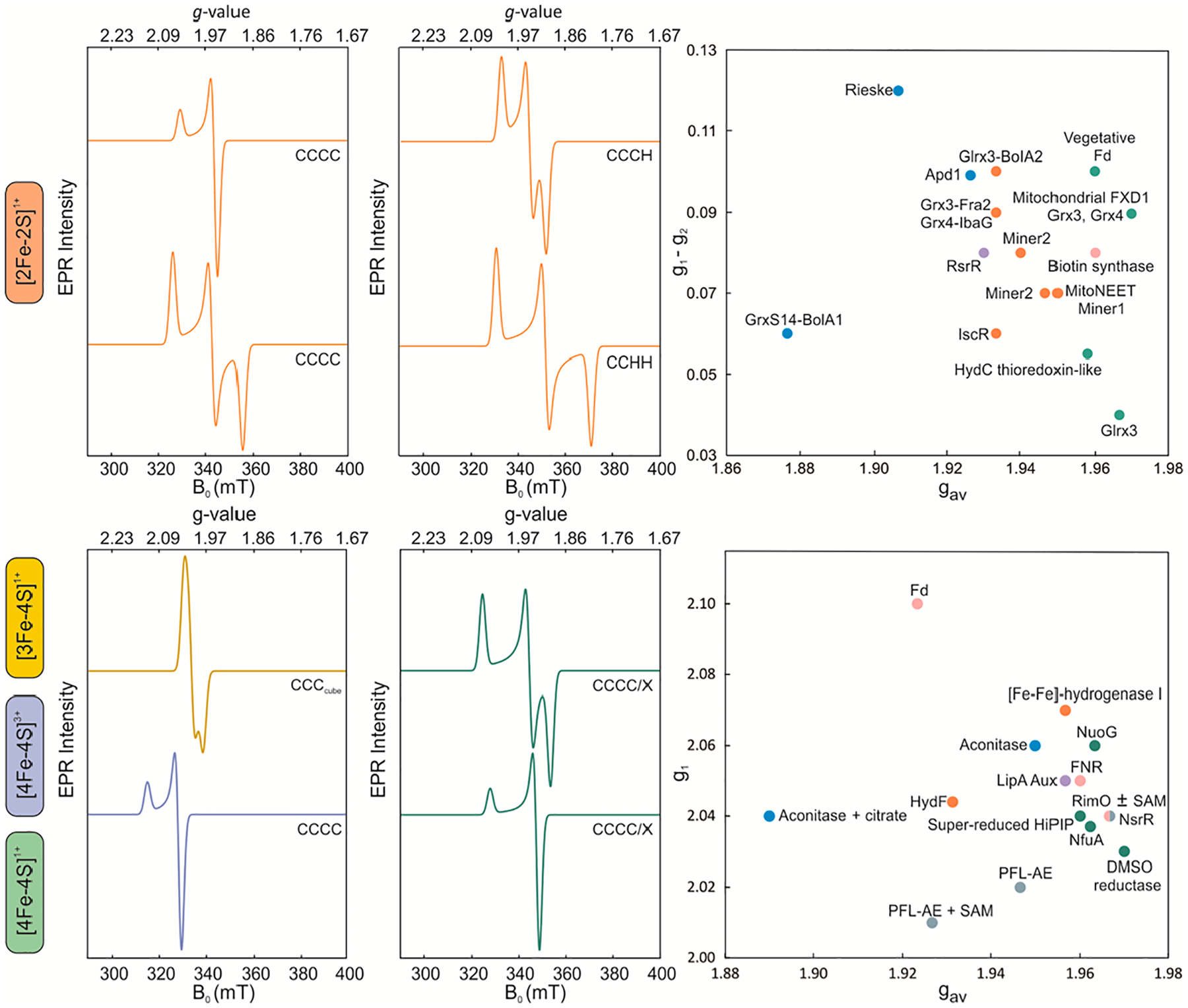
Characteristic X-Band CW EPR spectra of Fe-S clusters. (Top) The g_1_–g_2_ versus g_av_ plot is for the [2Fe-2S]^1+^ clusters. The ligand sets of CCCC and CC(GSH)_2_ are shown in green, CCCH and CC(GSH)H in orange, CCCR in pink, CCHH and C(GSH)HH in blue, and CCHE in purple circles. (Bottom) The ligand sets of CCCC are shown in green, CCCH in orange, CCCD in pink, CCCS in purple, CCC(H_2_O) and CCC(citrate) in blue, and CCC(H_2_O)-RS and CCC(citrate)-RS in grey circles. The spectra are simulations and compilations of the EPR parameters collected in Table 2 and the associated references with Table 2.

[2Fe-2S]^1+^ clusters exhibit EPR spectra with ${\text{g}}_{\text{av}}=\left(\frac{g1+g2+g3}{3}\right)<2$ and g-anisotropy that are particularly sensitive to changes in the ligation at the valence-localized Fe^2+^ site and provide a good indicator of the chemical nature of cluster ligands (Table 1, Figure 2). [2Fe-2S]^1+^ clusters ligated by four cysteine thiolates or a combination of cysteine and glutathione thiolates are typically characterized by g_av_-values in the range of 1.95–1.96, while the g-anisotropy of the spectrum can be either rhombic or axial [49,59,77]. Replacement of one of the thiolates with a histidine at the reducible Fe site leads to a decrease in the overall g_av_ that is now between 1.95 and 1.93 [34,54,55,78]. [2Fe-2S] clusters with a CCCH ligand set have been reported in mitoNEET and its related proteins Miner 1 and Miner 2, the transcription factor IscR, and glutaredoxin heterocomplexes with BolA-like proteins, in which the histidine ligand is provided by the BolA partner [29,35,51–53,59]. The spectra are, in most reported cases (if not all), rhombic. The coordination of two histidines to the valence localized Fe^2+^ site causes a further decrease in the average g-values that now fall within 1.88–1.92. CCHH ligated [2Fe-2S] clusters have been identified in the prototypical Rieske electron transfer proteins, glutaredoxin heterocomplexes with BolA-like proteins, and the cytosolic yeast protein Adp1 [29,36–38,53,59,77].

While the decrease in the g_av_-value can be used to infer mixed-ligation in [2Fe-2S]^1+^ clusters, it is not diagnostic of the chemical nature of the protein-based ligand, especially as other types of coordination are possible. In *E. coli* biotin synthase, one of the cysteinyl thiolates is replaced by the amine of arginine, resulting in a g_av_ = 1.95 [58]. In the *Streptomyces venezuelae* transcription regulator RsrR, one of the Fe ions is coordinated by a cysteine and a glutamate, while the second Fe site is coordinated by a cysteine and a histidine [60]. The EPR spectrum of RsrR is rhombic with a g_av_ = 1.93, resembling CCCH ligated [2Fe-2S] clusters rather than the CCHH ligated [2Fe-2S] ones, presumably because the glutamate ligand to the Fe^3+^ ion results in a negligible contribution to g-anisotropy. Cumulatively, the combination of relaxation properties, g_av_, and g-anisotropy allows for reliable inference of the chemical nature of coordinating ligands for the valence localized [2Fe-2S]^1+^ clusters. The precise nature of the cluster ligands should be, however, confirmed by site-directed mutagenesis.

#### 2.2.2. [3Fe-4S] Clusters

[3Fe-4S] clusters are paramagnetic in their oxidized 1+ form with an overall ground spin state that depends on the metallocofactor geometry [76,79,80]. Cuboidal [3Fe-4S]^1+^ clusters have an S = 1/2 state, are fast relaxing species with an optimal detection temperature of 10 K, and exhibit a signal with small anisotropy and g_av_~2.01 (Figure 2) [39,62,79]. Linear [3Fe-4S]^1+^ clusters have a ground spin state of S = 5/2 and are characterized by a signal at g~9, along with additional resonances at 4.3 and 4.15 that are often detected up to 40 K [81,82]. Although they were initially produced from the cuboidal forms of trinuclear clusters either by partial unfolding or oxidative conditions, they have recently been identified in the GciS, a protein in the viral capsid of giant viruses [25]. The one-electron reduced [3Fe-4S]^0^ state is also paramagnetic but has an integer spin ground state (S = 2), rendering it EPR-silent [1]. Such Fe-S cluster states are amenable to CW EPR but require a parallel-mode resonator to allow for the detection of transitions between integer spin eigenstates.

#### 2.2.3. [4Fe-4S] Clusters

[4Fe-4S] clusters attain four redox states, with the 2+ and 0 forms having integer ground states of S = 0 and S = 2, respectively (EPR-silent), while the 3+ and 1+ states have half-integer ground states (EPR-active). The [4Fe-4S]^3+^ cluster state is the one-electron oxidized form of a [4Fe-4S]^2+^ cluster and is exclusively found in electron transfer ferredoxins that are designated as high-potential proteins (HiPIPs) [41,63,64]. [4Fe-4S]^3+^ clusters exhibit an S = 1/2 ground state and are characterized by an axial signal with g_av_ between 2.06 and 2.10, marking these two properties as distinctive traits of this type of cluster and allowing for their unequivocal identification (Figure 2). On the other hand, one-electron reduction of the diamagnetic [4Fe-4S]^2+^ state yields the [4Fe-4S]^1+^ form that most commonly attains an S = 1/2 electronic configuration, although higher spin states (3/2 and 7/2) or a statistical mixture thereof can be observed in biological Fe-S clusters [42,43,68,73,83–85].

Low-spin [4Fe-4S]^1+^ clusters are typified by a g_av_ < 2 (similar to [2Fe-2S]^1+^ clusters) and exhibit both axial and rhombic EPR spectra (Figure 2) [42]. [4Fe-4S]^1+^ clusters are best detected at 10 K and generally exhibit faster relaxation rates than [2Fe-2S]^1+^ clusters, making the detectable temperature range a partially distinguishing parameter between these two types of polynuclear Fe-S clusters. For [4Fe-4S]^1+^ clusters with all-cysteinyl coordination, the g_av_ is almost invariably 1.96 (Table 1). Substitution of one of the cysteinyl thiolates with a histidine can either decrease the overall g_av_ to ~1.93, as observed in *Thermotoga neapolitana* HydF [69], or leave the g_av_ unchanged, as seen in the *Clostridium acetobutylicum* [FeFe] Hydrogenase I [68] or the distal cluster of [Ni-Fe] hydrogenase [62]. Similar observations have been made for the replacement of one of the cysteines with an aspartate; the *Bacillus subtilis* regulatory proteins NsrR [72] and FNR [70,71] exhibit a g_av_ close to 1.96, whereas the *Pyrococcus furiosus* Fd exhibits a downshift to 1.92 [73]. Although a lower g_av_ may indicate a non-thiolate ligand, the substitution effect may be, in many cases, masked because of the extensive valence delocalization within the tetranuclear core that does not exert appreciable changes on the electronic structure that are detectably reflected in the g_av_ values. Non-thiolate ligation, however, can often yield higher spin states due to the more asymmetric geometric environment and the induced site differentiation of the Fe ions that makes these states more energetically favorable (Table 1).

[4Fe-4S]^1+^ clusters with solvent ligands in the position of the fourth cysteine, such as in aconitase and the radical SAM (RS) clusters, exhibit S = 1/2 signals that are essentially indistinguishable from those of CCCC coordinated clusters [40,74,75]. The unique Fe site can interact with substrates, and while the addition of citrate to aconitase dramatically changes the spectrum, the addition of SAM to RS clusters has variable effects with respect to g-value shifts, line broadenings, or intensity variance. Although EPR spectra may be used to probe substrate binding, the reorganizational ability and delocalization extent in [4Fe-4S] clusters may skew coordination changes due to atypical ligation or small molecule binding. Nevertheless, shifts in the g-values, stabilization of higher spin states, and line broadenings can serve as diagnostic markers of altered ligation.

### 2.3. Mössbauer Spectroscopy

The discovery and characterization of Fe-S clusters are strongly intertwined with ^57^Fe Mössbauer spectroscopy. The first published report in 1968 by Moss et al. [86] laid the groundwork for the application of Mössbauer spectroscopy for the characterization of Fe-S clusters. Although the exact chemical structure of the Fe-S cofactors was not known at that time, it was shown that (i) in dinuclear clusters, valences are largely trapped, while in tetranuclear clusters, valences are greatly delocalized, (ii) Fe ions of the same oxidation state can be chemically distinct, and (iii) the spectra can be used to infer electronic configuration and number of Fe species in a protein. Despite the infancy of the application of Mössbauer spectroscopy on Fe-S cluster-containing proteins, the study by Moss et al. demonstrated its tremendous potential for understanding the electronic structure of these polynuclear centers.

^57^Fe Mössbauer spectroscopy is a sensitive technique for probing the chemical environment of Fe ions in Fe-S clusters, as it measures small changes in the energy levels of ^57^Fe nuclei [1,76,80,87]. The simplest ^57^Fe Mössbauer spectrum is a quadrupole doublet that consists of two equal-area peaks. The parameters that characterize a quadrupole doublet are (1) Isomer shift δ (mm/s), which is the distance of the centroid of the doublet relative to zero velocity, and (2) Quadrupole splitting ΔE_Q_ (mm/s), which is the velocity scale separation between the peaks of the doublet. The isomer shift is a measure of electron density at the nucleus and can be used to infer oxidation state, spin state, and ligand covalency. In general, the effect of the oxidation state on the isomer shifts in Fe-S clusters follows the trend δ(Fe^3+^) < δ(Fe^2.5+^) < δ(Fe^2+^) [76,87]. The quadrupole splitting ΔE_Q_ is representative of the local symmetry of the electric field gradient around the ^57^Fe nucleus and reflects deviations from spherical symmetry. For Fe-S clusters, ΔE_Q_ typically follows the trend for the isomer shift δ for the formal oxidation state and is a good metric for changes in the ligand environment of a ^57^Fe nucleus [76,86].

Mössbauer spectra of paramagnetic Fe-S clusters are complex due to magnetic hyperfine interactions but provide detailed information about the spin state, magnetic couplings, and electron delocalization. At 4.2 K, the Mössbauer spectra of paramagnetic Fe-S clusters are magnetically split because the rate of electronic spin relaxation is slower than the ^57^Fe Larmor precession frequency (‘slow relaxation regime’). At T ≥ 80 K, however, Mössbauer spectra collapse into quadrupole doublets because the electronic spin relaxation is now faster than the ^57^Fe Larmor precession frequency (‘fast relaxation regime’) [76,86]. Mössbauer parameters can be temperature-dependent due to the second-order Doppler shift; therefore, different Fe-S clusters should be referenced at the same temperature [86,87]. Although the electronic configuration and hyperfine structure of Fe-S clusters can be elucidated by measurements at variable field and temperatures, in this study, we utilize high-temperature spectra to illustrate the effects of cluster type and coordination in the Mössbauer parameters. Because for some systems, no information for both temperature ranges exists, we report the Mössbauer parameters at 4.2 K.

#### 2.3.1. [2Fe-2S] Clusters

**Figure 3.**
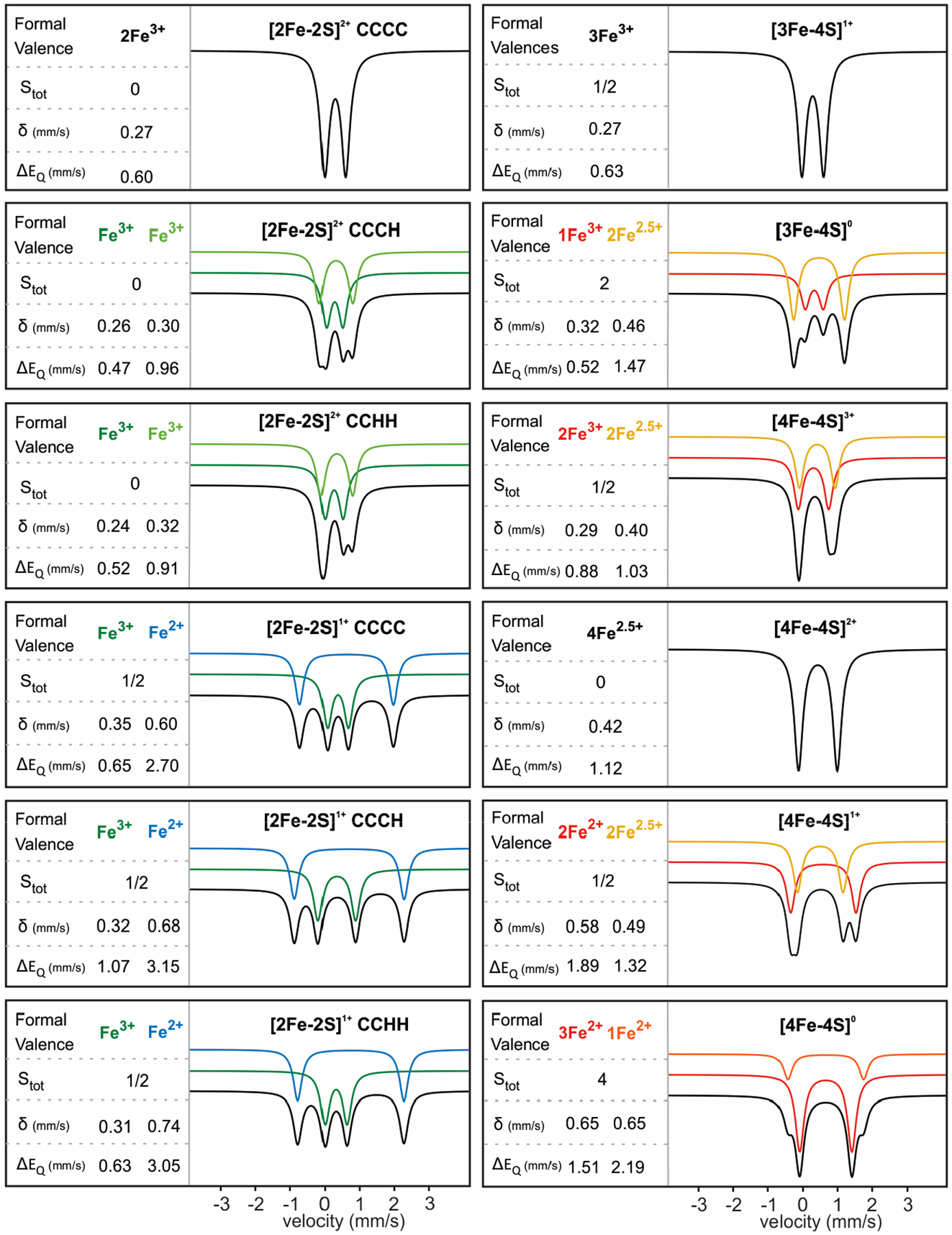
Characteristic Mössbauer spectra and parameters of [2Fe-2S], [3Fe-4S], and [4Fe-4S] clusters in their different oxidized states. The overall fit is shown in black; the individual quadrupole doublets (subcomponents) are shown in the colors corresponding to the formal valence of the specific Fe site or Fe pair. The spectra are simulations of parameters that have been adapted from references [1,34–38,40,48,51,58,77,79–83].

[2Fe-2S]^2+^ clusters consist of two AF-coupled high-spin Fe^3+^ ions (S = 5/2) and have an S = 0 ground state. All cysteinyl ligated [2Fe-2S]^2+^ clusters typically exhibit a single quadrupole doublet with δ = 0.27 mm/s and ΔE_Q_ = 0.60 mm/s, as both Fe^3+^ ions are chemically equivalent [49,88,89]. While the doublet can be asymmetric and better fit with two doublets (often referred to as ‘nested doublets’), in all cases studied to date, the average measured isomer shift for CCCC ligated [2Fe-2S]^2+^ clusters is ~0.27 mm/s (Figure 3).

Substitution of one cysteine with a histidine (CCCH) results in Mössbauer spectra that have two quadrupole doublets (Figure 3). The observed site differentiation stems from the greater electron delocalization exerted by the aromatic imidazole ring of the histidine, inducing the observed chemical inequivalence of the Fe^3+^ sites. The parameters of the cysteinyl ligated Fe^3+^ are similar to those of the ferric sites in CCCC ligated [2Fe-2S]^2+^ clusters with a small decrease in the quadrupole splitting (δ = 0.26 mm/s, ΔE_Q_ = 0.47 mm/s), while the mixed-ligated Fe^3+^ ion has upshifted δ = 0.30 mm/s and ΔE_Q_ = 0.96 mm/s [34].

Coordination by two cysteines and two histidines (CCHH) has been observed in [2Fe-2S] clusters in Rieske proteins, and their Mössbauer spectra further reflect the additional thiolate substitution [36–38]. The bis-cysteinyl-ligated Fe ion has δ = 0.24 mm/s and ΔE_Q_ = 0.52 mm/s, while the bis-histidyl-ligated Fe ion has δ = 0.32 mm/s and ΔE_Q_ = 0.91 mm/s. Overall, in both CCCH and CCHH ligated [2Fe-2S]^2+^ clusters, the histidine coordination appears to exert a larger quadrupole splitting for the associated Fe ion that is rationalized considering covalency differences between Fe-S and Fe-N bonds, causing a d-orbital charge anisotropy. [2Fe-2S]^2+^ clusters with mixed non-histidyl-ligation (with the exception of that in biotin synthase [58]) are characterized by two nested doublets (Table 2). The observed site-differentiation can thus conservatively serve as a good reporter of changes in the coordination environment of the associated Fe ion and inform on the presence of mixed ligation.

[2Fe-2S]^1+^ clusters consist of an AF-coupled Fe^3+^- Fe^2+^ pair with a paramagnetic (S = 1/2) ground state. CCCC ligated [2Fe-2S]^1+^ clusters exhibit high-temperature Mössbauer spectra that consist of two doublets corresponding to the ferric (δ = 0.35 mm/s and ΔE_Q_ = 0.65 mm/s) and ferrous (δ = 0.60 mm/s and ΔE_Q_ = 2.70 mm/s) sites, respectively (Figure 3). CCCH ligated [2Fe-2S]^1+^ clusters, such as the one in mitoNEET, have Mössbauer spectra that exhibit significant upshifts in δ = 0.68 mm/s and ΔE_Q_ = 3.15 mm/s for the ferrous site (to which histidine is a ligand), but only slight upshifts in δ = 0.32 mm/s and ΔE_Q_ = 1.07 mm/s for the ferric site (Table 2). Coordination of a second histidine to the ferrous site of the dinuclear core, such as in the case of Rieske proteins, leads to greater upshifts in its parameters with δ = 0.74 mm/s and ΔE_Q_ = 3.05 mm/s, while the ferric ion exhibits parameters similar to those of all thiolate ligated clusters (δ = 0.31 mm/s and ΔE_Q_ = 0.63 mm/s). Assuming that the geometry and spin state of the ferrous ion are retained, the value of the isomer shifts follow the trend δ(S) < δ(N) < δ(O) [76,87]. Thus, Mössbauer parameters of [2Fe-2S]^1+^ clusters are more sensitive reporters of mixed ligation when compared to those of the [2Fe-2S]^2+^ clusters, owing to the greater electron density of the ferrous ion (Table 2).

#### 2.3.2. [3Fe-4S] Clusters

Cuboidal [3Fe-4S]^1+^ clusters consist of three ferric ions in a spin-frustrated configuration with an S = 1/2 ground state [91]. The high-temperature Mössbauer spectrum exhibits a single quadrupole doublet with δ = 0.27 mm/s and ΔE_Q_ = 0.63 mm/s, parameters characteristic of high-spin Fe^3+^ ions. Linear [3Fe-4S]^1+^ clusters have an overall ground spin state of S = 5/2 and exhibit an δ = 0.28 mm/s. The ΔE_Q_ parameter remains undetermined due to the slow electronic relaxation [91]. One-electron reduction to the [3Fe-4S]^0^ form results in an electronic configuration that consists of a localized Fe^3+^ ion (S = 5/2) that is AF-coupled to the delocalized Fe^2.5+^-Fe^2.5+^ pair (S = 9/2) resulting in an S = 2 ground state. The Mössbauer spectrum consists of two doublets with a 1:2 relative ratio and the following parameters: δ(Fe^3+^) = 0.32 mm/s and ΔE_Q_(Fe^3+^) = 0.52 mm/s and δ(Fe^2.5+^) = 0.46 mm/s and ΔE_Q_(Fe^2.5+^) = 1.47 mm/s.

#### 2.3.3. [4Fe-4S] Clusters

[4Fe-4S] clusters attain up to four different oxidation states, with 3+, 2+, and 1+ being biologically accessible. The most oxidized [4Fe-4S]^3+^ form consists of a Fe^3+^-Fe^3+^ pair that is AF-coupled to a delocalized Fe^2.5+^-Fe^2.5+^ pair to yield an S = 1/2 ground state. The high-temperature Mössbauer spectrum exhibits two equal-intensity quadrupole doublets for the two Fe pairs with parameters: δ(Fe^3+^) = 0.29 mm/s and ΔE_Q_(Fe^3+^) = 0.88 mm/s and δ(Fe^2.5+^) = 0.40 mm/s and ΔE_Q_(Fe^2.5+^) = 1.03 mm/s [1,76,87]. One electron reduction yields the diamagnetic [4Fe-4S]^2+^ form that is made up of two AF-coupled valence-delocalized Fe^2.5+^ pairs. The Mössbauer spectrum is characterized by a single quadrupole doublet as all four Fe^2.5+^ ions experience a similar chemical environment and are thus spectroscopically indistinguishable. The Mössbauer parameters have more positive values, reflecting the more reduced state of the cofactor with δ = 0.42 mm/s and ΔE_Q_ = 1.12 mm/s. One electron reduction of the [4Fe-4S]^2+^ form yields the [4Fe-4S]^1+^ form, in which a diferrous pair is AF-coupled to the delocalized mixed-valence pair, yielding a half-integer state that is typically S = 1/2, although higher spin states are also accessible. The [4Fe-4S]^1+^ high-temperature Mössbauer spectrum exhibits two quadrupole doublets; the one for the Fe^2.5+−^Fe^2.5+^ pair has similar parameters to those of the cognate pairs in [4Fe-4S]^2+^ clusters (δ = 0.49 mm/s and ΔE_Q_ = 1.32 mm/s), while the diferrous pair is characterized by upshifts in the isomer shift and quadrupole splitting (δ = 0.58 mm/s and ΔE_Q_ = 1.89 mm/s). Although not biologically relevant, the all-ferrous state [4Fe-4S]^0^ has been observed with parameters δ = 0.65 mm/s and ΔE_Q_ = 1.51–2.19 mm/s (Figure 3) [92].

While the extensive electron delocalization and structural flexibility of the core of [4Fe-4S] clusters allow for a high degree of thermodynamic plasticity, these properties also skew covalency effects imposed by the incorporation of non-thiolate ligands. Therefore, non-conventional Fe-S cluster ligands do not exert appreciable changes in the Mössbauer spectra, often rendering ligand changes in tetranuclear clusters undetectable. A notable exception is the addition of substrates, such as citrate to aconitase or SAM to RS clusters, in which the “valence delocalization paradigm” in [4Fe-4S]^2+^ clusters breaks, due to changes in the number of ligands and asymmetry, promoting partial valence trapping in one of the mixed-valent diiron pairs [40,74,80]. The resulting Mössbauer spectra exhibit a site-differentiation because one of the Fe^2.5+^ ions attain a more ferrous character that is accompanied by an increase in the isomer shift and quadrupole splitting of the Fe site that interacts with the small molecule.

### 2.4. Supplementary or Alternative Approaches

#### 2.4.1. Chemoproteomics

While mass-spectrometric approaches have been of limited applicability to Fe-S cluster-containing proteins due to the acid lability of their cofactors, new advances have spawned powerful toolsets for the characterization of these metalloclusters. Brun and coworkers have broken new grounds utilizing native mass-spectrometry, a technique that allows for accurate mass detection of whole proteins containing an intact Fe-S cluster [93,94]. This approach utilizes isotopically labeled proteins (i.e., ^57^Fe, ^34^S, etc.) and allows for an unambiguous determination of the type of Fe-S cluster and its thermodynamic properties. Although isotopic labeling is not required, it makes the assignment of these cofactors easier. Native mass-spectrometry has been conducive to determining the type and oxidation status of Fe-S clusters in transcription factors, such as the NsrR and RsrR regulatory repressors, and in combination with time-resolved measurements has elucidated kinetics of Fe-S cluster conversion in the Fumarate and Nitrate Reduction (FNR) transcription factor [94]. Native mass-spectrometry is less sample demanding when compared to Mössbauer spectroscopy, requiring lower concentrations of Fe-S clusters (20–200 μM), but can be more intricate as it additionally calls for an apo form of the protein and samples of separate isotopic enrichment (i.e., ^57^Fe, ^34^S, and a mixture thereof).

Although the establishment of the Fe-S cofactor type can be arduous, perhaps the most challenging task is to determine the protein-based coordination of the metallocofactor. Identification of Fe-S cluster ligands is often carried out by structural and spectroscopic studies on wild-type and variant proteins generated by alanine or serine substitutions. However, the substitution of single and multiple candidate residues does not always abolish the metallocofactor, as the protein can reorganize to accommodate solvent-based ligands. The identity of the Fe-S cluster cysteine ligands can be exploited by chemoproteomic techniques that take advantage of the reactivity of these residues with IA-alkyne labels. This method, first applied by Weerapana and coworkers [95], relies on the fact that cysteines ligated to the Fe-S clusters on a holoprotein minimally react with the IA-alkyne, whereas in the apoprotein, these same residues are significantly more accessible. A difference in the extent of labeling between the holo and apoproteins (either with the same or different alkylating agent) can aid in the identification of Fe-S cluster ligands, in particular in cases in which amino acid substitutions do not allow for the unequivocal assignment of cysteine ligands.

#### 2.4.2. Pulse EPR

The above chemoproteomic techniques are only applicable to the identification of cysteines but unsuitable for other Fe-S cluster ligands. The second most common coordinating amino acid is histidine, but its ligation is not discernible from CW EPR spectra (see Section 2.2). Histidine coordination can be determined by time-domain EPR techniques (pulsed EPR), such as the electron spin echo envelope modulation (ESEEM) or the hyperfine sublevel correlation spectroscopy (HYSCORE) [69]. Such experiments can resolve the hyperfine interaction of the nuclear spin of the ^14^N (or isotopically labeled ^15^N) of the histidine with the unpaired electronic spin of the Fe-S cluster, unequivocally assigning a histidine as a Fe-S cluster ligand. Employing pulse EPR and mutagenesis, the exact residue can be confirmed by carrying out the same experiment on a protein variant containing an alanine in the position of the histidine, in which the signals from the nitrogen nucleus will be absent. The same approach is not only pertinent for resolving histidine coordination but also applicable for other N-based ligands. The presence of arginine as the fourth ligand in the [2Fe-2S] cluster of biotin synthase was validated by ESEEM spectroscopy, in which the hyperfine coupling of the arginine ^14^N that was present in the spectra of the WT enzyme was subsequently lost by substitution of this residue with a methionine [57].

#### 2.4.3. NMR Spectroscopy

In combination with Mössbauer and EPR, NMR spectroscopy can aid in the identification of the Fe-S cluster type and oxidation state and elucidate the magnetic coupling patterns in polynuclear Fe-S clusters [96,97]. Because NMR spectroscopy is performed in solution, it can also detect weak and transient protein–protein interactions that are invisible to other techniques. In this direction, NMR spectroscopy breaks new ground in the study of Fe-S cluster trafficking between the scaffold and target proteins, providing unique structural information on the atomic level about the chemical nature of these processes [57]. Although paramagnetic relaxation enhancement has been a limiting factor in the applicability of NMR spectroscopy to paramagnetic Fe-S clusters, the field has greatly advanced to allow for the utilization of NMR for gaining insight into the electronic structure of these cofactors in their different oxidation states [96–98]. An additional advantage of NMR is the identification of nearby hydrogen atoms, therefore providing remarkable chemical information about the hydrogen bonding networks surrounding these clusters. Moreover, NMR can yield dynamic structural information that is inaccessible to Mössbauer and EPR.

Although paramagnetic centers in proteins will cause line broadening due to paramagnetic relaxation, there are several (new and old) avenues to circumvent this issue and allow for interrogating protein/ligand-protein interactions as well as providing structural restraints. Fe-S clusters have been substituted by Ga^3+^, which is diamagnetic, allowing for the solution structure of putidaredoxin to be obtained and the interaction interface of the ferredoxin–thioredoxin–reductase FTR with its electron partner ferredoxin to be mapped [99–101]. Additionally, the structure of the IscU scaffold protein was solved by substituting the Fe-S cluster with the diamagnetic Zn^2+^ [102]. Recent efforts by Banci et al. have extended the applicability of paramagnetic NMR when combined with double and triple resonance experiments to allow for the spectroscopic characterization of Fe-S clusters and their ligands, as well as obtaining structural information [97]. New pulse sequences have been developed that minimize quenching of signal intensity due to the presence of a paramagnetic cofactor, setting the stage for the characterization of metalloproteins by solution-state NMR [96–98]. Overall, NMR spectroscopy has expanded its suite of tools that now enable its utilization for the study of Fe-S cluster proteins.

## 3. Identification of a Fe-S Cluster in the Hepatitis Virus HBx Oncoprotein

### 3.1. The Hepatitis Virus HBx

Hepatitis B virus (HBV) [103] causes acute and chronic hepatitis, and the latter is a major risk factor for the development of hepatocellular carcinoma (HCC) [104,105]. HCC is the third most common cause of cancer mortality and the fifth most common cancer worldwide [106]. At the core of the HBV infectivity lies the 17-kDa regulatory oncoprotein X (HBx) [107–109]. HBx is the etiological agent of HCC, and during tumorigenesis, it acts as a transcriptional transactivator of a multitude of cellular and viral factors [104,108]. Although the exact contributions of HBx to virus replication remain obscure, it is a multifunctional regulator that mediates HBV-associated pathogenesis by altering gene transcription, signaling pathways, genotoxic stress responses, protein degradation, DNA repair, cell proliferation, and apoptosis [107,110].

Despite the abundance of reported functions, the mechanistic role of HBx as a viral oncogene is poorly defined. The major challenge has been the lack of any direct structural or functional information about HBx on its own or in the context of the proteins it targets. One of the key limitations in this respect has been that HBx is sparingly soluble when expressed in insect, human, and bacterial cells and is often isolated from inclusion bodies unless expressed as a chimera with solubility tags or protein partners. Recently, heterologously expressed HBx has been reported to bind divalent transition metal ions, invoking a fundamental and previously ignored connection between the presence of a metallocofactor, protein structure, and activity. Therefore, advancing our knowledge of the molecular underpinnings behind HBV-related pathogenesis requires establishing the chemical nature of the HBx metallocofactor and the structure/function relationships supporting its role in viral-induced pathogenesis.

### 3.2. Spectroscopic Identification of the Cofactor

Although the HBx sequence is rich in cysteines, it does not harbor any recognizable metal binding motifs, nor does it possess significant sequence homology to any annotated proteins, and thus does not allow for inference of the type of the putative metallocofactor. Heterologously produced HBx (tethered to various solubility tags) and isolated aerobically exhibits optical spectra consistent with [2Fe-2S] clusters, demonstrating that HBx is a Fe-S-binding protein. When isolated anaerobically, however, the optical spectra of HBx more closely resemble those of [4Fe-4S] clusters, suggesting that the [2Fe-2S] cluster form may be a product of oxidative degradation [23]. Mössbauer spectroscopy has confirmed that the Fe-S cluster in HBx exists as a mixture of these two forms, the ratio of which is dependent on the expression and isolation conditions.

**Figure 4.**
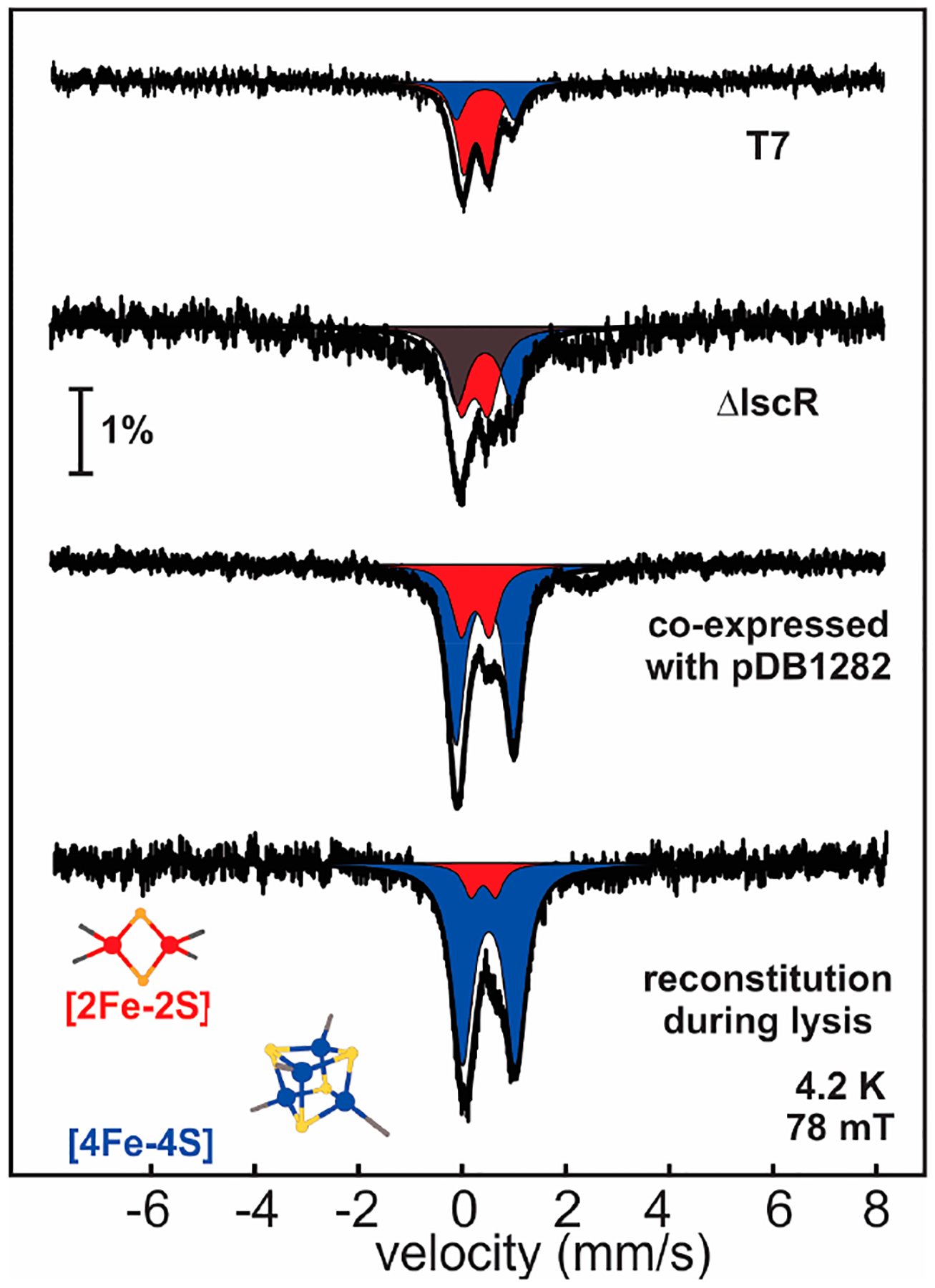
Mössbauer spectra of MBP-HBx isolated from *E. coli* cells under different expression conditions. The spectra were recorded at 4.2 K and in the presence of a small external field (78 mT), applied parallel to the γ-beam. The experimental spectra are shown with vertical black bars, the fit for the [2Fe-2S]^2+^ clusters with a red-shaded quadrupole doublet and the fit for the [4Fe-4S]^2+^ clusters with a blue-shaded quadrupole doublet. The data have been adapted by reference [111].

Owing to the fact that Fe-S clusters are notoriously O_2_-sensitive and degrade to smaller Fe-S fragments or decompose completely, HBx was anaerobically isolated from cells under different expression conditions (Figure 4). Expression of MBP-HBx in E. coli without overexpression of the ISC machinery yields a 4:1 ratio of [2Fe-2S] to [4Fe-4S] clusters. Expression either in ΔIscR E. coli strain (that lacks the IscR repressor) or co-expression with the pDB1282 plasmid [15] encoding for the ISC machinery increases the amount of Fe-S cluster enrichment as well as the ratio of assembled [4Fe-4S] clusters. These results demonstrated that (a) the native form of the Fe-S cofactor in HBx is a tetranuclear cubane [4Fe-4S] cluster, and (b) the observed inability to successfully assemble a [4Fe-4S] cofactor may be due to the high level of expression of MBP-HBx and/or the marked O_2_ sensitivity of the cofactor during the course of protein isolation. A method that proved effective in attaining a homogeneously enriched protein in [4Fe-4S] clusters was the co-expression of HBx with pDB1282, followed by reconstitution during lysis under anaerobic conditions. Therefore, identification of the biologically relevant form of Fe-S cofactors, in particular those that deviate from prototypical forms due to O_2_ sensitivity, ligation, or stability, may require additional steps of optimization to ensure a protein is homogeneously loaded with intact Fe-S clusters of the correct type.

### 3.3. Whole-Cell Mössbauer Studies to Establish the Physiological Fe-S Cluster Form in HBx

**Figure 5.**
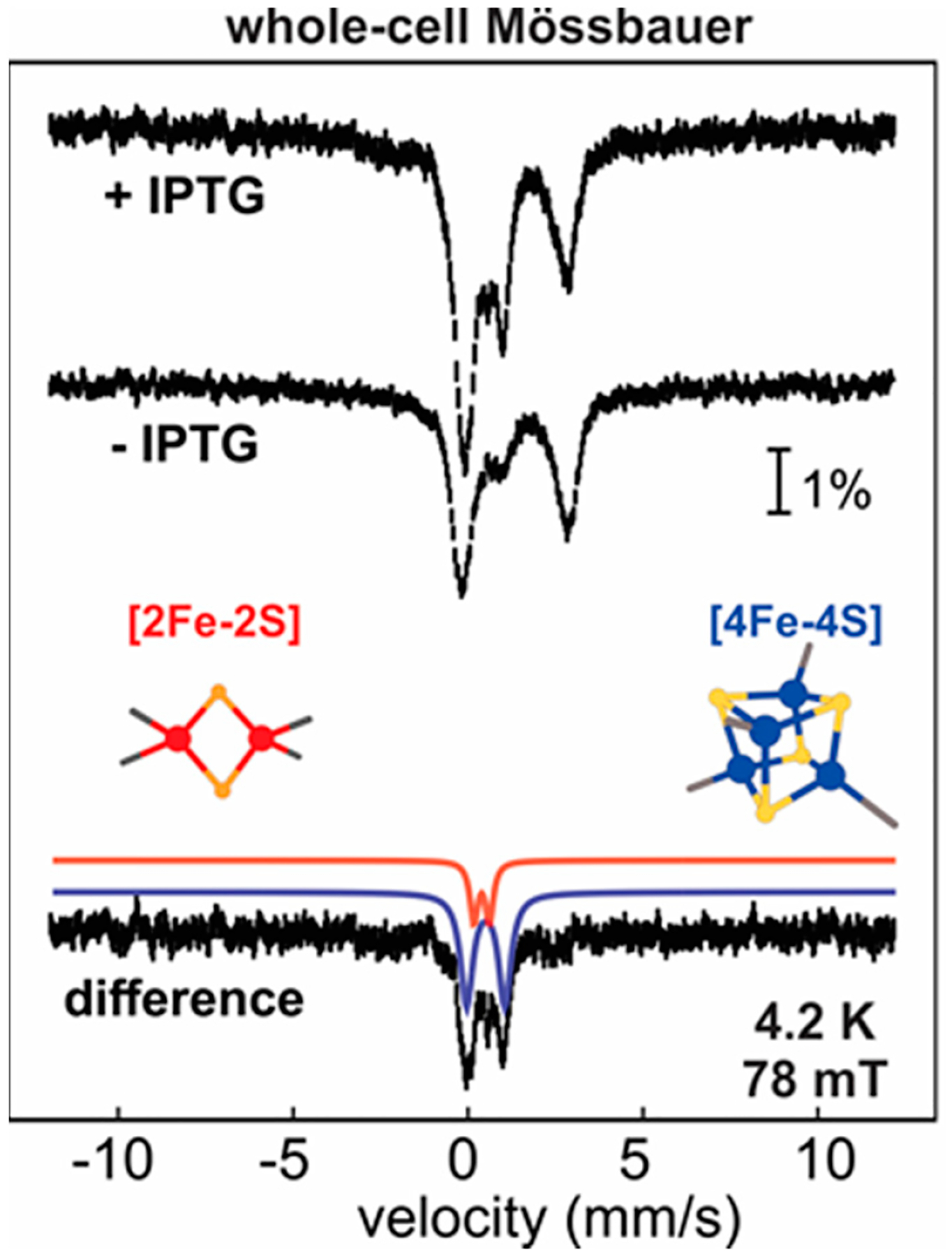
Whole-cell Mössbauer spectra of the cells expressing the MBP-HBx in T7 express cells in the presence and absence of IPTG. The experimental spectra are shown with vertical black bars, the fit for the [2Fe-2S]^2+^ clusters with a red shaded quadrupole doublet and the fit for the [4Fe-4S]^2+^ clusters with a blue shaded quadrupole doublet. The data have been adapted by reference [111].

Mössbauer experiments on intact cells carrying the MBP-HBx plasmid and grown in the presence of ^57^Fe were carried out to assess the distribution of different cluster forms when the protein is expressed alone in *E. coli* cells. For comparison, spectroscopy was performed on samples with and without IPTG induction to allow for the subtraction of unwanted contributions of endogenous Fe-containing *E. coli* proteins. The spectra show a 2:1 ratio of [4Fe-4S]:[2Fe-2S] clusters under cytoplasmic conditions (Figure 5) if HBx is not coexpressed with pDB1282 [15]. These results demonstrate that *E. coli* is unable to keep up with the demand for Fe-S clusters, resulting in the incorporation of lower nuclearity Fe-S types. Whole-cell Mössbauer, therefore, provides a valuable approach to track Fe-S cofactors in whole cells and interrogate the chemical nature of the cofactor in cellulo, obviating any artifacts resulting from protein isolation [112–114]. This method is also applicable when coexpressing with pDB1282 or in mammalian cells, followed by the addition of cysteine and ^57^Fe in the culture medium. Whole-cell experiments provide additional avenues for correlating oxidative stress and cofactor status in HBx-expressing (bacterial or mammalian) cells, with and without its reported protein targets. The proposed studies should be applicable in mammalian liver cells (i.e., HepG2, Huh7) transfected with HBx to confirm the in vivo chemical nature of the metallocofactor.

### 3.4. Redox Transformations of the HBx Fe-S Cluster

Two observations make the chemical identity of the HBx metallocofactor intriguing; it can be a [2Fe-2S] cluster or a [4Fe-4S] cluster, depending on the redox conditions, and the [2Fe-2S] form can undergo a reductive transformation that affords formation of [4Fe-4S] clusters. This reductive conversion was first observed by EPR spectroscopy and later confirmed by Mössbauer spectroscopy [23]. Although the aerobically isolated HBx contains [2Fe-2S] clusters, reduction with sodium dithionite for 20 min did not yield the expected paramagnetic [2Fe-2S]^1+^ cluster form, but rather an EPR signal with principal g-values and relaxation properties reminiscent of those of [4Fe-4S]^1+^ clusters [23]. The reductive conversion was also monitored in a time- and reducing equivalent-dependent manner by Mössbauer and EPR spectroscopy. Conversion of the pre-existing [2Fe-2S] clusters occurred without exogenous addition of Fe or sulfide and was complete within 30 min. The number of reducing equivalents required, the appearance of a transient [2Fe-2S]^1+^ cluster signal in the EPR spectra at early reaction times, and the absence of accumulation of a transient Fe^2+^ signal in the Mössbauer spectra collectively supported that the observed dithionite-induced transformation is best described by a reductive coupling mechanism. In such a scheme, two one-electron reduced [2Fe-2S]^1+^ clusters, presumably from two different monomers of HBx, are reductively coupled to yield the higher nuclearity [4Fe-4S] cofactor. Currently, it is not known whether this reductive rearrangement to afford a higher nuclearity cofactor is physiologically relevant, but it is noteworthy that analogous transitions have primarily been observed in Fe-S cluster donor proteins, such as IscU and IscA [115–117]. This molecular similarity implies that HBx may exhibit the potential to act as a rogue Fe-S cluster donor, a function that may be linked to HBx-mediated oncogenesis.

### 3.5. HBx Preferentially Binds a Fe-S Cluster and Not Zn

HBx was initially proposed to bind a Zn ion via three conserved cysteines and a histidine, four residues known to be important for the inactivation of the chromosome 5/6 complex (Smc5/6) that suppresses HBV transcription. In this pathway, HBx repurposes a ubiquitin ligase for the degradation of Smc5/6, and this function was suggested to be dependent on Zn binding [118]. Despite the depth of these seminal studies, there was no direct link between Zn binding and HBx function, while simultaneous substitution of all four proposed ligands did not abolish Zn content. In a subsequent study, cofactor incorporation in HBx was systematically examined under different metal supplementation conditions that demonstrated competitive binding of both metallocofactors, thus invoking a common binding site [23]. The ability of HBx to coordinate a redox-active [4Fe-4S] cluster challenges its purported Zn specificity and follows the paradigm of many proteins shown to bind Zn and Fe-S clusters in an often interchangeable manner. In addition, HBx has been attributed to cellular functions linked to ROS generation, which could be rationalized considering a redox-active Fe-S cofactor. The discovery that HBx is a Fe-S cluster protein resonates with the growing number of viral proteins initially considered to be Zn-dependent on the basis of a ZF CCCH motif but subsequently shown to coordinate Fe-S clusters. These are the catalytic subunit Nsp12 of the SARS-CoV-2 RNA-dependent polymerase [22], the Nsp13 helicase that associates with the RNA polymerase to afford replication of the viral genome [21], and the oncogenic protein sT of MCPyV that enhances DNA replication [24]. A very recent example is the low molecular weight protein GciS, which is the most abundant protein in the viral particle of *Megavirinae* giant viruses that coordinates a Fe-S cluster, for which the function is presently unknown [25]. The occurrence of Fe-S clusters in the viral proteome appears far more common than initially considered, and it is provoking to think that the incorporation of these cofactors must rely on the host Fe-S biosynthesis machinery, which is hoaxed into delivering these metalloclusters to viral recipients. For the case of HBx, the precise mechanism of cluster incorporation as well as its functional role remain enigmatic, but studies are under way to establish its recognition by Fe-S scaffold proteins and how it fosters HBx-mediated HBV pathogenesis on a molecular level.

## 4. Conclusions

Fe-S clusters are increasingly recognized as key cofactors in proteins across all domains of life and, more recently, viruses. From performing critical roles in the chemistry of central metabolism to serving as cryptic cofactors in oncogenic viral proteins, these metalloclusters are truly perplexing and omnipresent features of biology. Identification of Fe-S clusters, however, is often overlooked due to their inherent O_2_ sensitivity and unusual coordination patterns that make their inference solely on the basis of primary amino acid sequence challenging. The present review collects a detailed enchiridion of UV/VIS, EPR, and Mössbauer spectroscopies, three techniques that are mainstays in the study of these cofactors, and how these methods can be employed to deduce their nuclearity, oxidation, and spin states, as well as their ligation motifs. Additionally, we describe in brief other emerging techniques that provide unique information about the dynamics and electronic structure of these cofactors. In the end, we outline how the combination of UV/VIS, EPR, and Mössbauer spectroscopies resolved a multidecade conundrum by establishing that the oncogenic protein HBx from HBV harbors a redox-active Fe-S cluster.

## Figures and Tables

**Table 1.** EPR parameters of biologically relevant Fe-S clusters with different ligations that are included also in the Figure 2 of the Review. The first column consists of the ligation pattern motif, C is for cysteine, H for histidine, GSH for glutathionine, R for arginine, E for glutamate, D for aspartate, RS for Radical-SAM.

Coordination	Protein	*S_tot_*	(*g*_1_, *g*_2_, *g*_3_)^a^	*g*_av_	Temperature (K)^a,b^
**[2Fe-2S]** ^**1+**^					
**CCCC**	Mitochondrial FXD1 [48]	1/2	2.03, 1.94, 1.94	1.97	4–100
	Vegetative Fd [49]	1/2	2.05, 1.95, 1.88	1.96	4–100
	HydC thioredoxin-like [50]	1/2	2.01, 1.95, 1.92	1.96	40
**CC(GSH)**_**2**_	Glrx3 [51]	1/2	2.01, 1.97, 1.92	1.97	26
	Grx3 [52]	1/2	2.03, 1.94, 1.94	1.97	26
	Grx4 [53]	1/2	2.03, 1.94, 1.94	1.97	20
**CCCH**	mitoNEET [54,55]	1/2	2.01, 1.94, 1.90	1.95	5–60
	Miner1 [55]	1/2	2.01, 1.94, 1.90	1.95	20
	Miner2 [55]	1/2	2.00, 1.92, 1.90	1.94	20
	Miner2 [55]	1/2	2.01, 1.94, 1.89	1.95	20
	IscR [9,35]	1/2	1.99, 1.93, 1.88	1.93	20
**CC(GSH)H**	Glrx3–BolA2 [51]	1/2	2.01, 1.91, 1.88	1.93	26
	Grx3-Fra2^C^ [52,56]	1/2	2.01, 1.92, 1.87	1.93	4–70
	Glrx4-IbaG^C^ [53]	1/2	2.01, 1.92, 1.87	1.93	20
**CCCR**	Biotin synthase [57,58]	1/2	2.03, 1.95, 1.90	1.96	6–80
**CCHH**	Rieske [38]	1/2	2.02, 1.90, 1.80	1.91	4–100
	Apd1 [36]	1/2	2.01, 1.91, 1.86	1.93	10
**C(GSH)HH**	GrxS14-BolA1 [59]	1/2	2.02, 1.96, 1.65	1.88	10
**CCHE**	RsrR [60,61]	1/2	2.00, 1.92, 1.87	1.93	10
**[3Fe-4S]** ^**1+**^					
**CCC**_**cube**_	[NiFe] hydrogenase [62]	1/2	2.02, 2.00, 1.97	2.00	2–12
**CCC**_**linear**_	GciS [25]	5/2	9.10, 4.30, 4.15	5.85	4–40
**[4Fe-4S]** ^**3+**^					
**CCCC**	HiPIP [41,63,64]	1/2	2.12, 2.03, 2.03	2.06	4–20
**[4Fe-4S]** ^**1+**^					
**CCCC**	Super-reduced HiPIP [64]	1/2	2.04, 1.92, 1.92	1.96	4–40
	NuoG [65]	1/2	2.06, 1.94, 1.89	1.96	4–60
	DMSO reductase [66]	1/2	2.03, 1.94, 1.94	1.97	4–30
	NfuA [67]	1/2	2.04, 1.95, 1.90	1.96	5–35
**CCCH**	[FeFe]-hydrogenase I [68]	1/2	2.07, 1.93, 1.87	1.96	4–20
	[FeFe]-hydrogenase I [68]	3/2	5.60, 1.71, 1.31	2.87	4–30
	[FeFe]-hydrogenase I [68]	7/2	5.21, 5.13, 4.91	5.08	4–80
	HydF [69]	1/2	2.04, 1.90, 1.85	1.93	10
**CCCD**	FNR [70,71]	1/2	2.05, 1.94, 1.89	1.96	15
	NsrR [72]	1/2	2.04, 1.93, 1.93	1.97	10
	Fd [73]	1/2	2.10, 1.87, 1.80	1.92	4–15
**CCCS**	LipA Aux [74]	1/2	2.05, 1.91, 1.91	1.96	13
**CCC(H**_**2**_ **O)**	Aconitase [39]	1/2	2.06, 1.93, 1.86	1.95	13
**CCC(citrate)**	Aconitase + citrate [39]	1/2	2.04, 1.85, 1.78	1.89	13
**CCC(H**_**2**_ **O)-RS**	PFL-AE [40]	1/2	2.02, 1.94, 1.88	1.95	12
	RimO [75]	1/2	2.04, 1.93, 1.93	1.97	14
**CCC(SAM)-RS**	PFL-AE + SAM [40]	1/2	2.01, 1.89, 1.88	1.93	4–40
	RimO + SAM [75]	1/2	2.04, 1.93, 1.93	1.97	14

a The simulations correspond to representative spectra at X-Band microwave frequency;

b Temperature parameters without a defined range are measured at a specific temperature, with no temperature-dependent studies;

c The [2Fe-2S] cluster in the *S. cerevisae* Fra2-Grx3 heterodimers is more stable than in Grx3 homodimers, but the cluster in the *E. coli* Grx4-IbaG complex is less stable compared to Grx4 homodimers.

**Table 2.** Mössbauer parameters of different ligated [2Fe-2S]^1+^ clusters at 4.2 K.

System	Formal Valence	δ (mm/s)	|ΔE_Qav_| (mm/s)
[2Fe-2S]^2+^			
Rieske (CCHH) [38]	Fe^3+^	0.24	0.52
	Fe^3+^	0.32	0.91
mitoNEET (CCCH)	Fe^3+^	0.26	0.47
	Fe^3+^	0.30	0.96
IscR (CCCH) [35]	Fe^3+^	0.27	0.48
	Fe^3+^	0.30	0.72
Grx3 (CC(GSH)_2_) [52]	Fe^3+^	0.29	0.55
	Fe^2+^	0.29	0.76
Grx-Fra2 (CHXGSH) [52]	Fe^3+^	0.30	0.50
	Fe^2+^	0.32	0.82
IscU (CCCD) [90]	Fe^3+^	0.27	0.66
	Fe^3+^	0.32	0.94
Apd1/Aim32 (CCHH) [36]	Fe^3+^	0.24	0.54
	Fe^3+^	0.35	1.06
RsrR (CCHE) [60,61]	Fe^3+^	0.28	0.54
	Fe^3+^	0.29	0.76
Biotin synthase (CCCR) [57,58]	Fe^3+^	0.29	0.51
	Fe^3+^	0.29	0.51
[2Fe-2S]^1+^			
Rieske (CCHH) [38]	Fe^3+^	0.31	0.63
	Fe^2+^	0.74	3.05
mitoNEET (CCCH) [34]	Fe^3+^	0.32	1.07
	Fe^2+^	0.68	3.15
IscR CCCH [35]	Fe^3+^	0.33	1.09
	Fe^2+^	0.70	3.40
Apd1/Aim32 (CCHH) [36]	Fe^3+^	0.32	0.81
	Fe^2+^	0.75	3.16
